# Water bottle sign of pericardial effusion on chest radiograph

**DOI:** 10.11604/pamj.2020.37.360.27258

**Published:** 2020-12-21

**Authors:** Yee Li Xien, Sarvesh Seger

**Affiliations:** 1 School of Medicine, International Medical University, Clinical Campus Kluang, Kluang, Johor 86000, Malaysia

**Keywords:** Water bottle, pericardial effusion, chest radiograph

## Image in medicine

A 69-year-old lady diagnosed with congestive cardiac failure three years prior presented with worsening shortness of breath and bilateral leg swelling. She does not have a history of acute coronary syndrome, however an angiogram performed two years ago revealed a two-vessel disease. She also has hypothyroidism secondary to thyroidectomy. A cardiovascular examination revealed a raised jugular venous pulsation, displaced apex beat, muffled heart sounds, a pan systolic murmur heard loudest at the mitral region with radiation to the axilla and mid inspiratory crepitations over the lung bases with pitting oedema till the level of the mid-shin. These features are suggestive of an acute decompensation of congestive cardiac failure with mitral regurgitation. Her previous echocardiogram revealed an ejection fraction of 28% with global hypokinesia, dilated left ventricle and severe mitral regurgitation. A chest radiograph was done and revealed a very massive symmetrical cardio pericardial silhouette. This heart has a globular appearance, a flask or water bottle configuration with relatively smooth left and right cardiac contours. This is characteristic of pericardial effusion. The patient was treated with intravenous diuretics and supplemental oxygen along with the necessary supportive treatment of cardiac failure.

**Figure 1:**
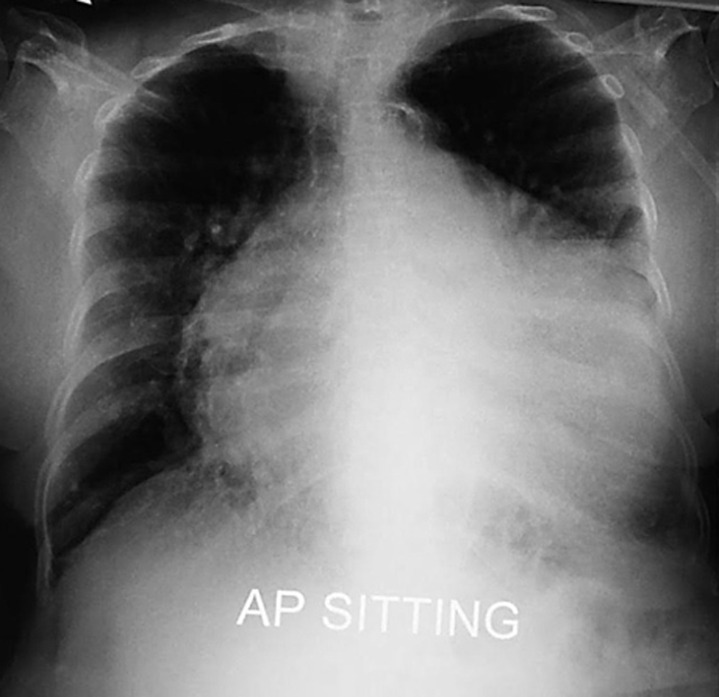
water bottle sign of pericardial effusion

